# Electroacupuncture Treatment Improves Neurological Function Associated with Regulation of Tight Junction Proteins in Rats with Cerebral Ischemia Reperfusion Injury

**DOI:** 10.1155/2014/989340

**Published:** 2014-06-10

**Authors:** Ya-min Zhang, Hong Xu, Hua Sun, Su-hui Chen, Fu-ming Wang

**Affiliations:** Department of Traditional Chinese Medicine, Peking Union Medical College Hospital (PUMCH), Peking Union Medical College (PUMC), Chinese Academy of Medical Sciences, Beijing 100730, China

## Abstract

Strategies to develop effective neuroprotective therapy to reduce brain damage and related behavioral deficits in stroke patients are of great significance. Electroacupuncture (EA), which derives from traditional Chinese medicine, may be effective as a complementary and alternative method for promoting recovery of neurological function and quality of life. Adult Sprague-Dawley rats were randomly divided into 3 groups: (1) sham, (2) middle cerebral artery occlusion (MCAO) model groups of 2 h MCAO followed by 1, 3, 5, or 7 d of reperfusion, and (3) EA groups of 2 h MCAO followed by 1, 3, 5, or 7 d of reperfusion. EA groups received EA therapy by needling at GV20 and left ST36. The results show that EA therapy improved the neurological function and reduced infarct volume, confirmed by modified neurological severity scores and TTC staining. Real-time PCR, immunohistochemistry, and western blot assay verified that EA upregulated the expression of tight junction (TJ) claudin-5, occludin, and zonula occluding-1 from 1 to 7 d after reperfusion. Our findings suggest that EA reduces brain damage and related behavioral deficits via upregulation of the TJ proteins.

## 1. Introduction

Strokes are either ischemic or hemorrhagic, with more than 80% of stroke cases caused by cerebral ischemia [1]. Although many advances have been made in the treatment of stroke, current therapies are lacking in effectiveness. There is a significant unmet need for developing novel and rational strategies aimed at reducing nervous system impairments caused by cerebral ischemia reperfusion injury (CIRI). Electroacupuncture (EA) was derived 2,500 years ago in ancient China and has been an alternative therapy complementing conventional medicine for stroke. Our previous studies demonstrated that EA at Baihui (GV20) and Zusanli (ST36) in rats after CIRI improves the neurological score and reduces the expression of MMP-9 and the inflammatory reaction in brains of rats [2, 3]. EA has recently been shown to accelerate rehabilitation of patients with brain ischemia. However, the potential mechanism is not fully understood; more evidence is needed before EA treatment is accepted clinically.

Reperfusion is necessary to restore blood flow and reduce neuronal damage caused by ischemia. However, brain damage even became worse after the blood flow is restored [4, 5]. The development of safe and effective agents to alleviate cerebral ischemia reperfusion injury will certainly improve the prognosis of ischemic stroke. Many pathological mechanisms, like inflammation, increase reactive oxygen species and brain edema, leading to blood-brain barrier (BBB) destruction. Postischemic reperfusion causes edema formation by targeting cerebral capillary endothelial cells, an important component of neuronal microenvironment called the neurovascular unit (NVU). Other components include astrocytes, pericytes, neurons, and extracellular matrix around the vessels [6]. These components together form the blood-brain barrier (BBB) that protects the brain from potentially toxic substances and regulates the passage of circulating molecules according to size and physical-chemical characteristics [7]. Tight junctions (TJs) are located between adjacent endothelial cells of the BBB and function as gates for molecular transportation through paracellular clefts [8]. TJ is a complex network of proteins including the transmembrane proteins occludins, claudins, and peripheral membrane protein family of zonula occludins (ZOs) and other molecules. TJs respond to cellular stimuli dynamically via disassembly, redistribution, degradation, and remodeling to maintain the structural and functional integrity of the BBB [9, 10]. ZO-1 acts as a crucial central regulator of the structural organization of the TJ. It is expressed around the vessels, especially at sites of endothelial cell-cell contact, suggesting that ZO-1 preserves the integrity of the BBB [11]. In fact, a decrease in ZO-1 increases brain edema [12]. Claudin-5 and occludin are localized at the leading edge of the brain microvascular cells and are the major protein components of the transmembrane. TJs act as significant roles in regulating the permeability of BBB [8, 13]. Studies show changes in ZO-1 and occludin expression which are consistent with BBB permeability changes and are related to BBB opening [14, 15]. Decreased mRNA and protein expression levels of ZO-1, claudin-5, and occludin are closely associated with BBB breakdown and edema in the ischemic brain [16] and also the neuroprotective effects of chemical agents against ischemic injury through the prevention of TJ protein downregulation [17].

In the present study, we evaluated how EA at GV20 and ST36 changed infarct volumes, neurological deficits, and expression levels of TJ-associated proteins ZO-1, claudin-5, and occludin following cerebral ischemia reperfusion within 7 d in Sprague-Dawley rats.

## 2. Materials and Methods

### 2.1. Animals

Adult male Sprague-Dawley (SD) rats (*n* = 90) weighing 230–250 g (Peking Union Medical College Hospital, Beijing, China) were housed in an environmentally controlled room at 22 ± 2°C, with a 12 h/12 h light/dark cycle, and the rats were allowed free access to food and water throughout the whole study. The study was approved by the Ethics Committees of Peking Union Medical College Hospital and Chinese Academy of Medical Sciences for the Care and Use of Laboratory Animals, and we made all efforts to minimize the animal suffering in the study.

### 2.2. Rat Model of Cerebral Ischemia Reperfusion

Focal cerebral ischemia was established in the rats based on the methods described by Longa et al. [18]. Briefly, rats were anesthetized with 10% chloral hydrate (100 g/0.3 mL, intraperitoneal injection). The right common carotid artery (CCA), external carotid artery (ECA), and internal carotid artery (ICA) were exposed. A 4–0 suture (diameter 0.26 mm) with a blunted tip coated with poly-L-lysine was gently advanced into the ICA through the ECA. The suture was advanced 18–20 mm (reaching the origin of the right middle cerebral artery) beyond the carotid artery bifurcation. To allow the reperfusion, the suture was slowly withdrawn after 2 h of middle cerebral artery occlusion (MCAO).

### 2.3. Grouping and Treatment

Rats were randomly divided into the following 3 groups: (1) sham, (2) middle cerebral artery occlusion (MCAO) model group of 2 h MCAO followed by 1, 3, 5, or 7 d of reperfusion, and (3) EA groups of 2 h MCAO followed by 1, 3, 5, or 7 d of reperfusion. The sham group rats received all surgical procedures, but the suture was not advanced into the ICA. No treatments were conducted in the sham and the MCAO model groups.

Rats in the EA groups received the first EA treatment after the withdrawal of the suture after 2 h of MCAO and then received EA treatment 20 min session once daily. GV20 is located on the top of the head at the intersection of the middle sagittal line and the connection of two ear apexes. ST36 is located 3 individual cun below genu and one fingerbreadth before the anterior crest of the tibia. The rats in the EA groups were needled at GV20 and ST36 with disposable, sterile acupuncture needles. Two electrodes (Changzhou Wujin Great Wall Medical Instrument Co., Ltd., China) were attached for acupuncture and continuous-wave stimulation at a frequency of 2 Hz (intensity 1 mA) for 20 min. The 20-minute session once daily EA regiment for the intervention group was chosen based on our previously described reports [2, 19] and from stroke patients in clinical practice [20].

### 2.4. Neurological Functional Evaluation

We evaluated neurological function in the rats (*n* = 9) at 1, 3, 5, and 7 d postreperfusion using the modified neurological severity scores (mNSS) [21] by an observer with no prior knowledge of the groups and treatments. The mNSS was composed of motor, sensory, balance beam test, and reflex tests and graded from 0 to 18. A higher mNSS score correlates with more severe injury (Table 1).

### 2.5. Infarct Volume Assessment

Rats (*n* = 3) were narcotized by an intraperitoneal injection of 10% chloral hydrate (100 g/0.3 mL) and sacrificed by decapitation 7 d after reperfusion to evaluate the volume of cerebral infarction. Brains were quickly removed and chilled in −20°C refrigerator for 10 min, and five 2 mm consecutive coronal slices were made beginning from the anterior pole. The slices were put into a solution of 0.1% 2,3,5-triphenyltetrazolium chloride (TTC; Sigma, USA) in PBS for 30 min at 37°C in darkness before being transferred into 4% paraformaldehyde for 1 h. The infarct region appeared white, and the normal tissue was red. To account for edema and differential shrinkage resulting from tissue processing, the percentage of infarct volume was calculated as follows: [(VC−VL)/VC] ×100%; VC represents the volume of the control hemisphere, and VL is the volume of the noninfarct tissue in the lesion hemisphere [22]. The sections were photographed and infarct size measured using image analysis software (ImageJ, NIH) by an observer with no prior knowledge of the experiment.

### 2.6. Immunohistochemistry

We used immunohistochemical staining to detect distribution and expression of ZO-1, claudin-5, and occludin in rats after cerebral ischemia reperfusion. At 1, 3, 5, and 7 d post-MCAO reperfusion, rats (*n* = 6) were narcotized and perfused transcardially with 250 mL of saline followed by 250 mL of 4% paraformaldehyde solution. Brains were removed and fixed in 4% paraformaldehyde solution at 4°C for 72 h and then dehydrated and embedded in paraffin blocks. Coronal sections at the level of optic chiasma in the infarct region were cut into 3 *μ*m thick pieces. Briefly, brain sections were deparaffinized and hydrated with decreasing concentrations of alcohol and then incubated for 15 min in 1% Triton X-100 to disrupt the cell membrane. The sections were incubated with 3%   H_2_O_2_ and 5% normal goat serum at room temperature for 30 min each. Brain sections were incubated overnight at 4°C with rabbit polyclonal anti-ZO-1 antibody (diluted 1 : 150; Zymed, USA), goat polyclonal anti-claudin-5 antibody (diluted 1 : 50; Santa Cruz Biotechnology, USA), and rabbit polyclonal anti-occludin antibody (diluted 1 : 150; Abcam, UK) and then the remaining procedures accorded with the standard procedures. Brain sections were photographed (Leica DM4000, Germany) and analyzed using image analysis software (Image J, NIH) for semiquantitative tests of MMP-2, AQP4, and AQP9 integrated optical density (IOD).

### 2.7. Quantitative Real-Time Polymerase Chain Reaction Analyses (qPCR)

Expression levels of mRNA for ZO-1, claudin-5, and occludin in the rats (*n* = 3) were determined by qPCR. Total RNAs were extracted from the ischemic hemisphere using the RNeasy mini kit (Omega, USA) and reverse transcribed to cDNA. The reverse transcription (RT) reaction was amplified on a Bio-Rad CFX96 Detection System (Applied Biosystems, USA) using the Plexor One-Step qRT-PCR System (Promega A4021, USA). For ZO-1, claudin-5, and occludin, the following primers were used: ZO-1 forward: 5′-GATGAGCGGGCTACCTTATTGA-3′, reverse 5′-TTGGTCGGGAGATCGTGACTG-3′; claudin-5 forward: 5′-GGTGAGCATTCGGTCTTTAGC-3′, reverse 5′-TTGTGGTCCAGGAAGGCAGT-3′; occludin forward: 5′-TGGGACAGAGCCTATGGAACG-3′, reverse 5′-TTGGTCGGGAGATCGTGACTG-3′. The fold change of relative mRNA expression was determined using the 2^−ΔΔCt^ [23] method and using GADPH as an endogenous reference: GADPH forward: 5′-CACAGCAAGTTCAACGGCACAG-3′, reverse 5′-GACGCCAGTAGACTCCACGACA-3.

### 2.8. Western Blot Analysis

The ischemic hemispheres of the rats (*n* = 3) were homogenized in Radio-Immunoprecipitation Assay (RIPA) lysis buffer (Beyotime Biotechnology, China); proteins were obtained by centrifugation at 13,000 g for 15 min at 4°C. Supernatants were harvested and the protein concentration of each sample was determined by bicinchoninic acid (BCA) assay (Beyotime Biotechnology, China). For gel electrophoresis, samples were separated on 12% SDS-polyacrylamide gels; separated proteins were electrotransferred to polyvinylidene fluoride (PVDF) membranes (Millipore, USA). Membranes were blocked for 2 h with 5% nonfat milk (BD, USA) at room temperature and then were incubated overnight at 4°C with primary antibodies: rabbit polyclonal anti-ZO-1 antibodies (diluted 1 : 500; Zymed, USA), goat polyclonal anti-claudin-5 antibodies (diluted 1 : 200; Santa Cruz Biotechnology, USA), and rabbit polyclonal anti-occludin antibodies (diluted 1 : 300; Abcam, UK). After three washes, membranes were incubated for 2 h at room temperature with HRP-conjugated secondary antibody (1 : 5000; Jackson, USA). Detection was performed using an ECL kit (Millipore, USA). Blots were subsequently probed for *β*-actin (Santa Cruz Biotechnology, USA) as an internal control for normalization of protein loading. The intensities of the bands were measured using image analysis software (Labworks 4.6, China).

### 2.9. Statistical Analysis

All data were analyzed with the statistical analysis software SPSS (Statistical Package for the Social Sciences) 17.0 and are presented as the mean ± standard deviation (SD). Differences among multiple groups were analyzed using one-way ANOVA, followed by LSD* t*-test. Statistical significance was assumed in all cases if *P* < 0.05.

## 3. Results

### 3.1. EA Improved the Neurological Function in Focal Cerebral Ischemia Rats

**Figure 1:**
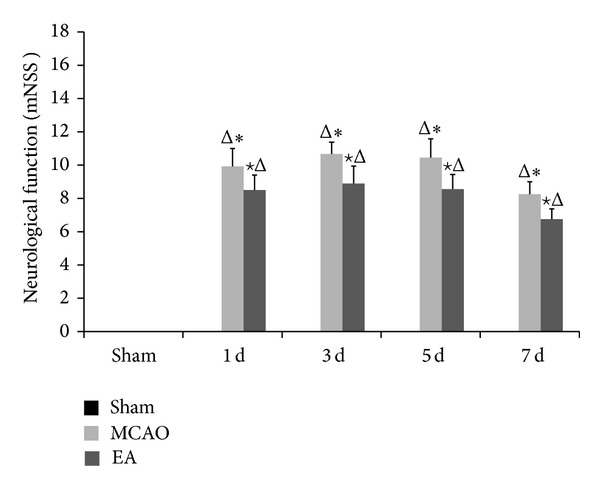
Neurological functional outcome. Measurement performed at 1, 3, 5, and 7 d after reperfusion using the mNSS. The score for the sham group on each day was 0. Rats receiving EA treatment showed significant improvement in neurological function compared with the MCAO model groups at the same time points. Data (*n* = 9) is represented as mean ± SD. ^Δ^ *P* < 0.05 versus the sham group, ^★^ *P* < 0.05 versus the MCAO group at the same time points, and *P < 0.05 versus the EA groups at the same time points.

We performed a neurological functional test to determine whether acupuncture treatment of CIRI rats regulates functional outcome. We assessed neurological function of the rats at 1, 3, 5, and 7 d after reperfusion using the mNSS. Rats receiving EA treatment showed significant improvement in neurological function compared to the MCAO model groups at the same time points (*P* < 0.05, *n* = 9) (Figure 1).

### 3.2. EA Reduced Infarct Volume in Focal Cerebral Ischemia Rats

**Figure 2:**
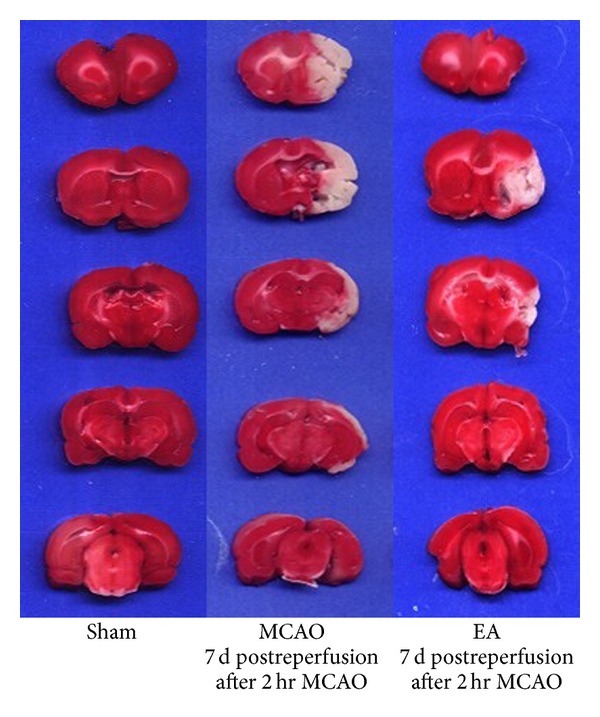
TTC staining of brain slices. Ischemic area is white; intact area stained red. Representative coronal sections of sham group, MCAO groups at 2 h MCAO followed by 7 d of reperfusion, and the EA groups of 2 h MCAO followed by 7 d of reperfusion.

**Figure 3:**
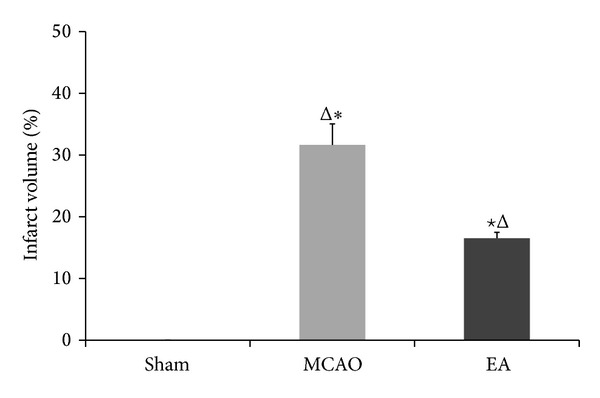
Quantification from TTC staining of infarct volume in focal cerebral ischemia rats of 7 d after reperfusion. The ischemic infarct volume was quantified using image analysis software. The infarct volume of the sham group was 0. The infarction induced by MCAO was significantly reduced by EA at GV 20 and ST 36 acupoints. Data (*n* = 3) is represented as mean ± SD. ^★^ *P* < 0.05 versus the MCAO groups at the same time points. *P < 0.05 versus the EA groups at the same time points.

TTC assay results show that the infarct regions remained white while the normal brain tissues were red (Figure 2). In the ischemic hemisphere, the cerebral infarction areas included frontoparietal cortex, temporal cortex, and lateral portion of neostriatum. EA treatment significantly decreased the volume of infarct regions at days 1 and 7 compared with untreated animals (*P* < 0.05, *n* = 3, Figure 3). These results support the neuroprotective effect of EA treatment against cerebral ischemic injury.

### 3.3. EA Changed the Distribution and Expression of mRNA and Protein of Tight-Junction Proteins ZO-1, Claudin-5, and Occludin in Focal Cerebral Ischemia Rats

**Figure 4:**
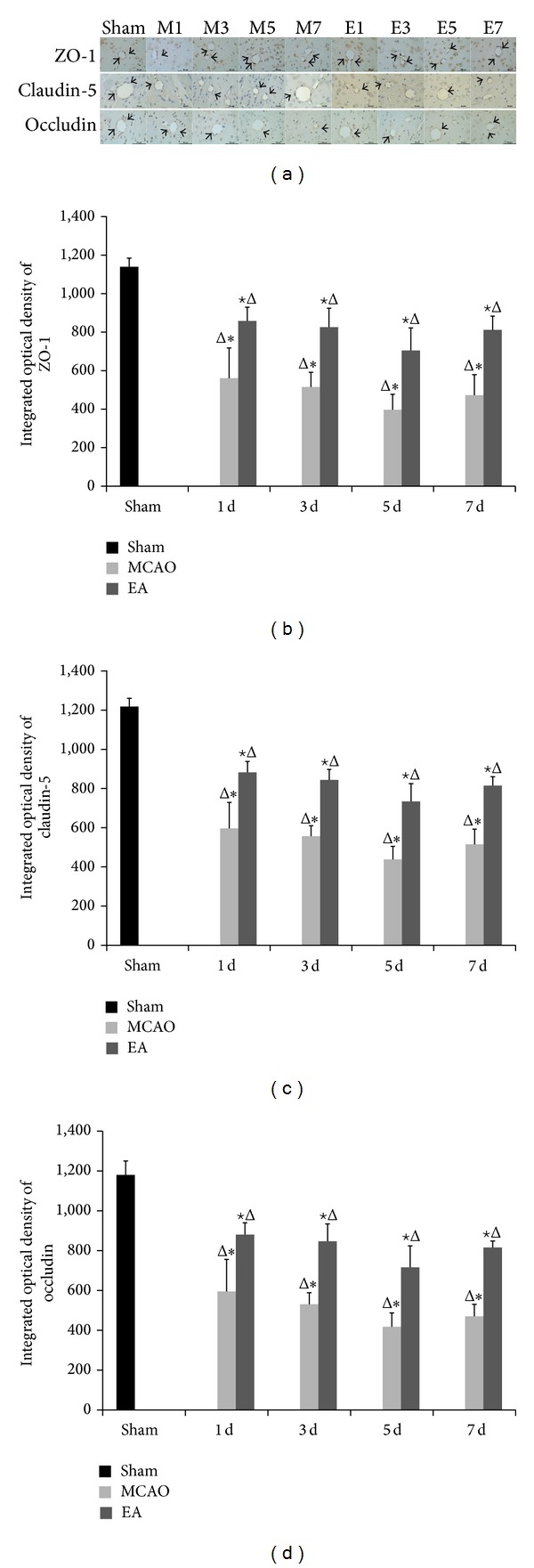
Effects of EA on the distribution and expression of ZO-1, claudin-5, and occludin on ischemic cerebral microvessels. (a) Representative immunohistochemistry stained tissue of the following groups: sham; M1–M7: MCAO groups after 1, 3, 5, and 7 d of reperfusion; and E1–E7: EA groups after 1, 3, 5, and 7 d of reperfusion. The integrated optical density of ZO-1 (b), claudin-5 (c), and occluding (d). Data (*n* = 6) are represented as mean ± SD. ^Δ^ *P* < 0.05 versus the sham group, ^★^ *P* < 0.05 versus the MCAO group at the same time points, and *P < 0.05 versus the EA groups at the same time points. Arrows show the immunoreactive positive area. Scale bar in* A* = 50 *μ*m (×400).

**Figure 5:**
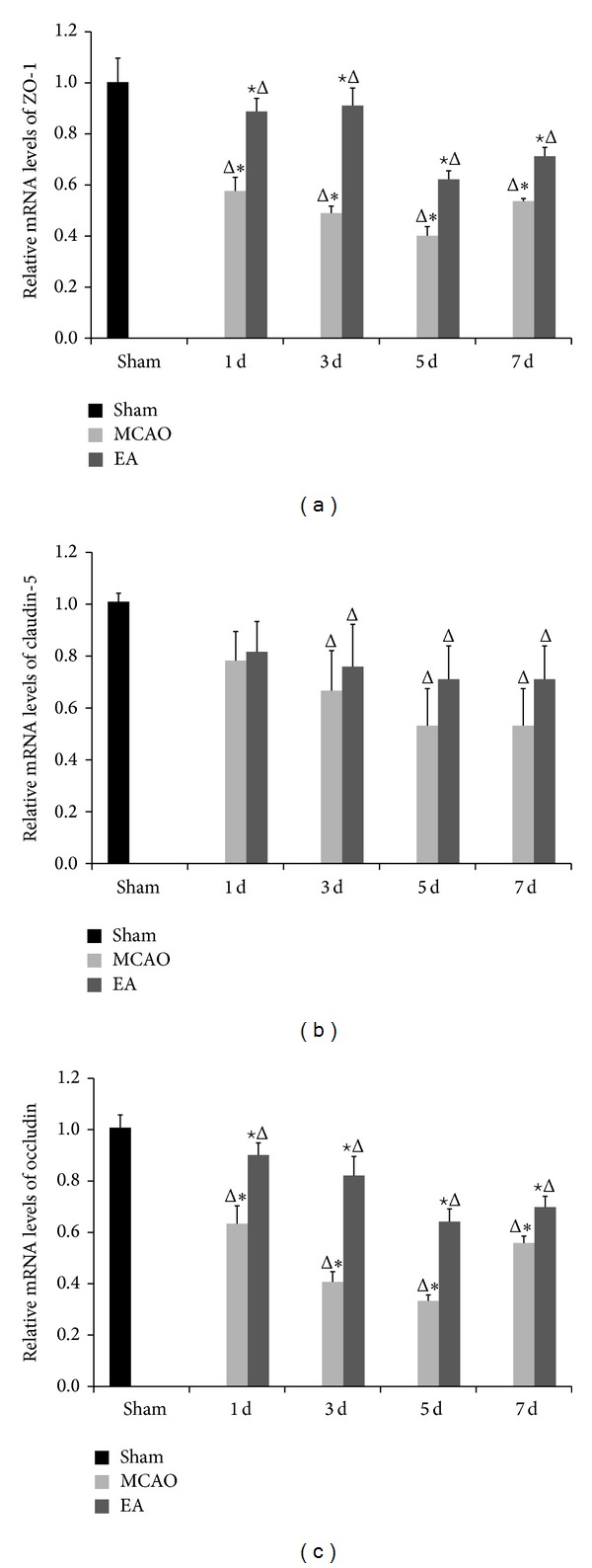
mRNA expression of ZO-1, claudin-5, and occludin analyzed by qPCR after EA. GAPDH served as endogenous reference. Experimental groups are sham; MCAO groups after 1, 3, 5, and 7 d of reperfusion; and EA groups after 1, 3, 5, and 7 d of reperfusion, respectively. The mRNA expression levels of ZO-1 (a), claudin-5 (b), and occluding (c) are shown. Data (*n* = 3) are represented as mean ± SD. ^Δ^ *P* < 0.05 versus the sham group, ^★^ *P* < 0.05 versus the MCAO group at the same time points, and *P < 0.05 versus the EA groups at the same time points.

We explored whether EA treatment maintains the BBB permeability via modulation of TJ proteins. We evaluated the distribution of ZO-1, claudin-5, and occludin by immunohistochemical staining, gene expression by qPCR, and protein expression western blot analysis. Immunohistochemistry staining results revealed that the expressions of ZO-1, claudin-5, and occludin were localized continuously on the cerebral microvessels in the sham group. In contrast, the expression of ZO-1, claudin-5, and occludin in the cerebral vascular structures in both MCAO and EA groups following reperfusion showed discontinuous staining and significant disruptions. Expression of ZO-1, claudin-5, and occludin decreased at days 1 to 5 d and then increased at day 7. The integrated optical density values of ZO-1, claudin-5, and occludin were increased significantly after EA treatment at 1, 3, 5, and 7 d after reperfusion compared to the MCAO groups (*P* < 0.05, Figure 4). qPCR results show mRNA expression levels of ZO-1 and occludin in MCAO groups and EA groups significantly declined compared to the sham group, but expression was significantly strengthened after EA treatment (*P* < 0.01, Figure 5).

**Figure 6:**
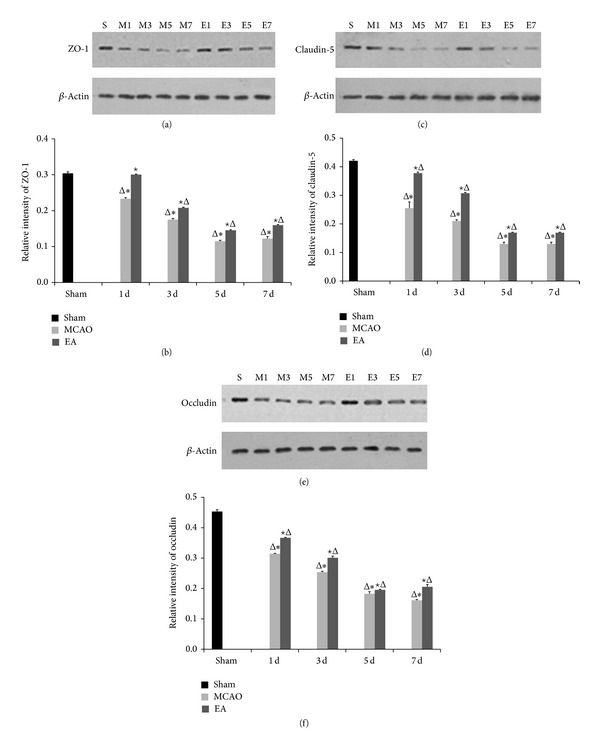
Protein expression levels of ZO-1, claudin-5, and occludin on ischemic cerebral microvessels after cerebral ischemia reperfusion. Western blots of (a) ZO-1, (c) claudin-5, and (e) occludin. From left to right: sham; M1–M7: MCAO groups after 1, 3, 5, and 7 d of reperfusion; and E1–E7: EA groups after 1, 3, 5, and 7 d of reperfusion. *β*-Actin served as a loading control. IDV of ZO-1 (b), claudin-5 (d), and occluding (f) are shown. Data (*n* = 3) is represented as mean ± SD. ^Δ^ *P* < 0.05 versus the sham group, ^★^ *P* < 0.05 versus the MCAO group at the same time points, and *P < 0.05 versus the EA groups at the same time points.

We analyzed changes in TJ protein expression induced by EA via western blot. Results show protein levels of ZO-1, claudin-5, and occludin in ischemic cerebral area were consistent with findings obtained from the immunohistochemical staining and qPCR (Figure 5). The protein expression levels of ZO-1, claudin-5, and occludin were significantly improved in the EA groups as compared with the MCAO groups at 1, 3, 5, and 7 d (*P* < 0.05, Figure 6). These data show EA therapy was effective on CIRI.

## 4. Discussion

In this study, we provide evidence showing EA at GV20 and ST36 in rats after CIRI reduced the ischemic brain damage in models of focal cerebral ischemia within 7 d of reperfusion. EA therapy was proved to reduce infarct volume and improve neurological functional outcome in acute ischemic stroke rats. We show that EA promotes BBB tightness by alleviating the disruption of the TJ-related proteins ZO-1, claudin-5, and occludin during the process of cerebral ischemia reperfusion. We argue that EA ameliorates focal cerebral ischemia reperfusion injury via modulation of TJ protein expression. Our findings suggest that EA should be considered complementary and alternative medicine for alleviating cerebral ischemia.

Reperfusion is necessary to reduce the neuronal damage caused by ischemia. However, reperfusion damage to the brain acts a crucial role in the pathophysiology of cerebral ischemic stroke [4, 5]. Clinically, ischemia reperfusion injury has become a challenge in ischemic cerebrovascular disease. Many pathological mechanisms are associated with reperfusion injury including inflammation, increase in reactive oxygen species, brain edema, BBB destruction, necrosis, and apoptosis.

The BBB consists of brain microvascular endothelial cells and astroglial cell end-feet that protect the brain from potentially toxic substances. It also regulates the passage of circulating molecules on the basis of size and physical-chemical characteristics. BBB disruption and brain edema formation both act important roles in the development of neurological dysfunction in acute and chronic cerebral ischemia. The structural and functional integrity of the BBB is maintained by interendothelial cell TJs. The BBB is constructed of TJs, including occludin and claudin-5, which form the endothelial barrier. TJs are a complex network of proteins and consisted of the transmembrane proteins (occludins, claudins, and junctional adhesion molecules) and peripheral membrane protein family of ZOs (ZO-1, ZO-2, and ZO-3). At the molecular level, the proteins ZO-1, claudin-5, and occludin are highly enriched at the TJ [8]. The TJs restrict the paracellular movement of solutes (water soluble and polar compounds) and small ions. ZO-1 is expressed around the vessels, especially at sites of endothelial cell-cell contact. ZO-1 acts as a crucial central regulator of the structural organization of the TJ and preserves the integrity of the BBB [11]. In fact, a decrease in ZO-1 coincides with an increase in cerebral edema [12]. The claudin family, specifically claudin-5, which is localized at the leading edge of brain microvascular cells, is important intercellular junction proteins and essential for the modulation of endothelial permeability. Previous reports prove that downregulation of claudin-5 is closely related to cerebral ischemia [8, 13, 24]. TJ protein occludin is critically involved in sealing the TJ [25], and its disruption alone is enough to cause functional changes of the TJ [26]. It also has been demonstrated that occludin is significantly reduced in CIRI rats [27]. Some chemical agents are neuroprotective against ischemic injury via the prevention of TJ protein downregulation [17]. Decreased expression of ZO-1, claudin-5, and occludin mRNA and protein is associated with BBB breakdown and edema in the ischemic brain [14, 16].

TJ protein downregulation, translocation, or phosphorylation is closely related with the BBB breakdown in focal cerebral ischemia reperfusion injury. However, there is limited data describing the relationship between TJ protein and recovery of ischemic stroke. Growing research on other central nervous system diseases, like multiple sclerosis, brain tumors, Parkinson's, and Alzheimer's, suggests that disruption of TJ integrity is associated with BBB dysfunctions. Various molecular mechanisms including downregulation, phosphorylation, degradation, or translocation of TJ proteins may work in the pathological process of these diseases [28–32].

We are in need of effective neuroprotective methods and rational strategies aimed at preventing CIRI and reducing impairments caused by it. Acupuncture is one of the major complementary and alternative therapies for stroke, and it has gained public support. According to traditional Chinese medicine, the body is an interconnected and self-contained system, relying on many factors to maintain a balanced and healthy state. Qi, blood, ying, and yang are vital matters among these elements. Qi flows in a system of meridians and functions to warm the body and protect it from illness. Blood and qi are closely related. Blood nourishes the qi and offers a material foundation keeping qi in the correct location. Blood contains the spirit, which is significant for the mental state. Yin and yang are two opposite sides of the substance in traditional Chinese, and they will not exist without each other. When yin and yang are functioning harmoniously they maintain a dynamic balance, with no clinical symptoms. However, in pathological conditions, the balance is interrupted.

GV20 belongs to the governor vessel, which may connect all yang vessels in the whole body. GV20 functions to regulate local qi and blood and modulates the balance between yin and yang. After being stimulated, GV20 makes the normal function of the body active, dispersing local yang. ST36 belongs to the stomach meridian, which is rich in both qi and blood. Thus, it is supposed as an acupoint for its role in the recovery of paralysis. The combined use of the two acupoints is effective in dredging the channels and collaterals, modifying the blood and qi and balancing yin and yang. EA therapy developed from traditional acupuncture, and compared with traditional acupuncture EA adds electrical stimulation to acupoints. Many researches focus on the mechanism of neuroprotective effect of the EA therapy. Many studies have certified that EA improves neurological function and neural apoptosis, promotes cell proliferation, and increases cerebral blood flow after stroke [33–35].

Available data indicate that EA pretreatment protects the integrity of BBB [36], reduces ischemic cerebral injury, and improves neurological outcomes [37–39]. This provides a clue that EA therapy may be a promising protective method for patients with high risk of cerebral ischemic disease. However, the function mechanisms of EA are complicated and all current studies provide limited information. Therefore, more research is needed.

In this study, we have demonstrated that the cerebral infarct size was significantly reduced with the treatment of EA. Additionally, we observed improvement in the functional outcome within 7 d of perfusion following 2 h of MCAO. These results suggest efficacy in EA-mediated neuroprotective function.

We explored the changes in the expression of mRNA and protein of ZO-1, claudin-5, and occludin at 1, 3, 5, and 7 d after reperfusion in EA-treated rats. The immunohistochemistry staining results revealed that the expressions of ZO-1, claudin-5, and occludin were localized on the cerebral microvessels in the sham group. The expressions of ZO-1, claudin-5, and occludin were decreased from d1 to d5 and were increased by d7 in both MCAO and EA groups with discontinuous staining and significant disruptions. The integrated optical density values of ZO-1, claudin-5, and occludin were increased significantly after EA. qPCR results revealed that mRNA expression levels of ZO-1 and occludin in MCAO groups and EA groups significantly declined with the progress of reperfusion compared to the sham group. However, expression was significantly enhanced after EA treatment. Western blot results support immunohistochemical staining and the qPCR results. The protein expression levels of ZO-1, claudin-5, and occludin were significantly improved in the EA groups as compared with the MCAO groups at 1, 3, 5, and 7 d. These data show that EA treatment was effective on focal cerebral ischemia reperfusion injury by affecting the stability of the TJ proteins.

To conclude, the present findings argue that EA application is critical to achieve a therapeutic effect for neuroprotection against ischemia/reperfusion injury. In addition, EA may preserve BBB function via regulation of TJ proteins, ZO-1, claudin-5, and occludin. However, the mechanism of the regulation remains unclear. It may involve a combination of phosphorylation modifications and internalization of TJs [28]. At the very least, this supports the need for further research to determine the clinical value of EA.

## Figures and Tables

**Table 1:** Description of the mNSS [21].

	Points
Motor tests	
Raising the tail of rat	3
1 forelimb flexion	
1 hindlimb flexion	
1 head moved >10° to vertical axis within 30 s	
Placing rat on floor	3
0 walking normally	
1 unable to walk straight	
2 circling toward paretic side	
3 falling down to paretic side	
Sensory tests	2
1 placing test	
2 proprioceptive test	
Beam balance tests	6
0 balance with steady posture	
1 grasp the side of beam	
2 hug beam with 1 limb falls down from beam	
3 on the basis of 2, but 2 limbs fall down from beam (>60 s)	
4 attempt to balance on beam but falls off (>40 s)	
5 on the basis of 4, but (>20 s)	
6 drop from the beam or with no action (<20 s)	
Reflex absence and abnormal movements	4
1 pinna reflex	
1 corneal reflex	
1 startle reflex	
1 seizures, myoclonus, and myodystony	
Maximum	18
